# Supplementary material for: An open-source framework for large-scale, flexible evaluation of biomedical text mining systems
Source: J Biomed Discov Collab. 2008 Jan 29;3:1. doi: 10.1186/1747-5333-3-1 (PMC2276192; doi:10.1186/1747-5333-3-1)
Supplement: Additional file 1 — Evaluation platform source code. The additional file contains the software evaluation framework discussed in this paper. The most current release can be downloaded from SourceForge.net [1]. See the accompanying README file for instructions on installation and use. Also included in the distribution is a collection of UIMA wrappers for some commonly used BioNLP tools and annotated corpora. [file 1747-5333-3-1-S1.zip › bionlp_uima_repository-1.0.1/docs/api/src-html/edu/uchsc/ccp/util/external/geniatagger/GeniaTaggerOutputFileParser.html]

```
001    package edu.uchsc.ccp.util.external.geniatagger;
002    
003    import java.io.BufferedReader;
004    import java.io.IOException;
005    import java.util.ArrayList;
006    import java.util.List;
007    
008    import edu.uchsc.ccp.util.nlp.GenericTextUtils;
009    import edu.uchsc.ccp.util.nlp.annotation.Annotator;
010    import edu.uchsc.ccp.util.nlp.annotation.TextAnnotation;
011    import edu.uchsc.ccp.util.nlp.mention.ClassMention;
012    import edu.uchsc.ccp.util.nlp.mention.ClassMentionTypes;
013    import edu.uchsc.ccp.util.nlp.mention.SlotMention;
014    import edu.uchsc.ccp.util.nlp.mention.SlotMentionTypes;
015    
016    public class GeniaTaggerOutputFileParser {
017    
018        private final boolean DEBUG = false;
019    
020        private BufferedReader br;
021    
022        private List<TextAnnotation> nextDocumentAnnotationList;
023    
024        private Annotator annotator;
025    
026        public GeniaTaggerOutputFileParser(String fileToParse) throws IOException {
027            annotator = new Annotator(new Integer(23), "GENIA tagger", "GENIA tagger", "TSJUII");
028    
029            br = GenericTextUtils.openReader(fileToParse);
030        }
031    
032        public boolean hasNext(String documentText) {
033            try {
034                String nextID = getNextID();
035                debug("Retrieved ID: " + nextID);
036                if (nextID == null) {
037                    note("Reached the end of the file... no more GENIA tagger output to return.");
038                    br.close();
039                    return false;
040                }
041                debug("About to extract annotations from [" + nextID + "] -- " + documentText);
042                extractAnnotationsForNextDocument(nextID, documentText);
043                debug("Finshed annotation extraction.");
044                
045            } catch (IOException e) {
046                e.printStackTrace();
047            }
048    
049            return true;
050        }
051    
052        public List<TextAnnotation> next() {
053            return nextDocumentAnnotationList;
054        }
055    
056        public void remove() {
057            throw new UnsupportedOperationException();
058    
059        }
060    
061        /* gets the token and chunk annotations from the input file. This method assumes that the first token of the sentence is on the very next line. */
062        private void extractAnnotationsForNextDocument(String documentID, String documentText) throws IOException {
063            String tokenLine;
064    
065            int startSearchingForTokenAtIndex = 0;
066    
067            nextDocumentAnnotationList = new ArrayList<TextAnnotation>();
068            List<String> chunkTags = new ArrayList<String>();
069    
070            int tokenCount = 0;
071            
072            while (((tokenLine = br.readLine()) != null) && (tokenLine.trim().length() > 0)) {
073                /*
074                 * parse the token line: it will take the form of word1 base1 POStag1 chunktag1\nword2 base2 POStag2 chunktag2
075                 */
076                
077                String[] toks = tokenLine.split("\\t");
078                if (toks.length != 4) {
079                    error("Expecting 4 tokens from line, but got: " + toks.length + "  LINE=" + tokenLine);
080                } else {
081                    String token = toks[0];
082                    String pos = toks[2];
083                    String chunkTag = toks[3];
084    
085                    /* add the chunk tag to the list so that chunks can be extracted later */
086                    chunkTags.add(chunkTag);
087    
088                    /* we must convert a token of '' and `` to " */
089                    if (token.equals("''") || token.equals("``")) {
090                        token = "\"";
091                    }
092    
093                    debug("Looking for token in text: " + token);
094                    
095                    try {
096                        TextAnnotation tokenAnnotation = GenericTextUtils.findTargetInText(token, documentText, startSearchingForTokenAtIndex,
097                                documentID, ClassMentionTypes.TOKEN, annotator, true).get(0);
098                        if (tokenAnnotation != null) {
099                            /* add the POS slot */
100                            SlotMention posSM = new SlotMention();
101                            posSM.setMentionName(SlotMentionTypes.TOKEN_PARTOFSPEECH);
102                            posSM.addSlotValue(pos);
103                            tokenAnnotation.getClassMention().addSlotMention(posSM);
104                            
105                            SlotMention tokenNumSM = new SlotMention();
106                            tokenNumSM.setMentionName(SlotMentionTypes.TOKEN_NUMBER);
107                            tokenNumSM.addSlotValue(Integer.toString(tokenCount));
108                            tokenAnnotation.getClassMention().addSlotMention(tokenNumSM);
109    
110                            startSearchingForTokenAtIndex = tokenAnnotation.getAnnotationSpanEnd();
111                            
112                            nextDocumentAnnotationList.add(tokenAnnotation);
113                        } else {
114                            error("Null annotation found for documentID: " + documentID + " token text: " + token);
115                        }
116                    } catch (IndexOutOfBoundsException iobe) {
117                        System.err.println("Caught index ob exception: looking for token: " + token + " in document: " + documentID);
118                    }
119                }
120                tokenCount++;
121            }
122    
123            /* check to make sure we have the correct number of annotations */
124            if (nextDocumentAnnotationList.size() != tokenCount) {
125                error("ERROR. Token count mismatch for document: " + documentID + ".  Expected: " + tokenCount + ", but got " + nextDocumentAnnotationList.size() + " annotations");
126            }
127            
128            /* create chunk annotations */
129            boolean openNP = false;
130            boolean openPP = false;
131            TextAnnotation npTA = null;
132            TextAnnotation ppTA = null;
133            int index = 0;
134    
135            for (String chunkTag : chunkTags) {
136    
137                /* if the chunk tag is a B-NP, we need to start a new NP (and close an old one if it is open). We also need to extend an open PP */
138                if (chunkTag.equals("B-NP")) {
139                    if (!openNP) {
140                        npTA = openNewPhraseAnnotation("NP", nextDocumentAnnotationList.get(index).getAnnotationSpanStart());
141                        openNP = true;
142                    } else {
143                        closeTextAnnotation(npTA, nextDocumentAnnotationList.get(index - 1).getAnnotationSpanEnd());
144                        npTA = openNewPhraseAnnotation("NP", nextDocumentAnnotationList.get(index).getAnnotationSpanStart());
145                        openNP = true;
146                    }
147                } else if (chunkTag.equals("I-NP")) {
148    
149                } else {
150                    if (openNP) {
151                        closeTextAnnotation(npTA, nextDocumentAnnotationList.get(index - 1).getAnnotationSpanEnd());
152                        openNP = false;
153                    }
154                }
155    
156                /* if you see an O tag, close any open phrases */
157                if (chunkTag.equals("O")) {
158                    if (openNP) {
159                        closeTextAnnotation(npTA, nextDocumentAnnotationList.get(index - 1).getAnnotationSpanEnd());
160                        openNP = false;
161                    }
162    
163                    if (openPP) {
164                        closeTextAnnotation(ppTA, nextDocumentAnnotationList.get(index - 1).getAnnotationSpanEnd());
165                        openPP = false;
166                    }
167                }
168    
169                index++;
170            }
171    
172        }
173    
174        private TextAnnotation openNewPhraseAnnotation(String phraseType, int spanStart) {
175    
176            TextAnnotation ta = new TextAnnotation();
177    
178            ta.setAnnotator(annotator);
179                ta.setAnnotationSpanEnd(spanStart + 1);
180                ta.setAnnotationSpanStart(spanStart);
181    
182            ClassMention cm = new ClassMention();
183            cm.setMentionName(ClassMentionTypes.PHRASE);
184    
185            SlotMention sm = new SlotMention();
186            sm.setMentionName(SlotMentionTypes.PHRASE_TYPE);
187            sm.addSlotValue(phraseType);
188            cm.addSlotMention(sm);
189            ta.setClassMention(cm);
190    
191    //        debug("Opening new phrase annotation: ");
192    //        ta.printAnnotation(System.err);
193    
194            return ta;
195        }
196    
197        private void closeTextAnnotation(TextAnnotation ta, int spanEnd) {
198                ta.setAnnotationSpanEnd(spanEnd);
199    
200    //        debug("Closing phrase annotation: ");
201    //        ta.printAnnotation(System.err);
202    
203            nextDocumentAnnotationList.add(ta);
204    
205        }
206    
207        /* gets the next id from the input file. This method assumes that the id is on the very next line of the buffered reader. */
208        private String getNextID() throws IOException {
209            String idLine = br.readLine();
210            if (idLine != null) {
211                String idStr = idLine.split("\\t")[0];
212                /* now skip the next line as it is blank */
213                String blankLine = br.readLine();
214                if (blankLine != null) {
215                    if (blankLine.trim().length() > 0) {
216                        error("Expecting blank line after ID but got: ID=" + idStr + " BlankLine=" + blankLine);
217                    }
218                } else {
219                    error("Expecting blank line after ID line - None found.");
220                }
221                return idStr;
222            } else {
223                return null;
224            }
225    
226        }
227    
228        
229        
230        
231        
232        private void note(String message) {
233            System.err.println("NOTE -- GeniaTaggerOutputFileParser: " + message);
234        }
235    
236        
237        private void error(String message) {
238            System.err.println("ERROR -- GeniaTaggerOutputFileParser: " + message);
239        }
240    
241        private void warn(String message) {
242            System.err.println("WARNING -- GeniaTaggerOutputFileParser: " + message);
243        }
244    
245        private void debug(String message) {
246            if (DEBUG) {
247                System.err.println("DEBUG -- GeniaTaggerOutputFileParser: " + message);
248            }
249        }
250    
251    }
```
